# Decreased neonatal pain response after vaginal-operative delivery with Kiwi OmniCup versus metal ventouse

**DOI:** 10.1186/s12884-017-1231-x

**Published:** 2017-01-31

**Authors:** E. A. Huhn, E. Visca, D. R. Vogt, S. von Felten, E. M. Tinner Oehler, C. Bührer, D. Surbek, R. Zimmermann, I. Hoesli

**Affiliations:** 1 Department of Obstetrics and Gynaecology, University Hospital Basel, Spitalstrasse 21, 4031 Basel, Switzerland; 2 Department of Obstetrics and Gynaecology, Cantonal Hospital Baselland, Liestal, Switzerland; 3 Clinical Trial Unit, University Hospital Basel, Basel, Switzerland; 4 Department of Paediatrics, Insel Hospital, University of Bern, Bern, Switzerland; 5 Department of Neonatology, Charité University Medical Center, Berlin, Germany; 6 Department of Obstetrics and Gynaecology, Insel Hospital, University of Bern, Bern, Switzerland; 7 Department of Obstetrics and Gynaecology, University Hospital Zurich, Zurich, Switzerland

**Keywords:** Bernese pain scale, EDIN pain scale, Metal cup, Kiwi OmniCup®, Vaginal operative delivery

## Abstract

**Background:**

Vaginal delivery, especially operative assisted vaginal delivery, seems to be a major stressor for the neonate. The objective of this study was to evaluate the stress response after metal cup versus Kiwi Omnicup® ventouse delivery.

**Methods:**

The study was a secondary observational analysis of data from a former prospective randomised placebo controlled multicentre study on the analgesic effect of acetaminophen in neonates after operative vaginal delivery and took place at three Swiss tertiary hospitals. Healthy pregnant women ≥35 weeks of gestation with an estimated fetal birth weight above 2000 g were recruited after admission to the labour ward. Pain reaction was analysed by pain expression score EDIN scale (Échelle Douleur Inconfort Nouveau-Né, neonatal pain and discomfort scale) directly after delivery. For measurement of the biochemical stress response, salivary cortisol as well as the Bernese Pain Scale of Newborns (BPSN) were evaluated before and after an acute pain stimulus (the standard heel prick for metabolic testing (Guthrie test)) at 48–72 h.

**Results:**

Infants born by vaginal operative delivery displayed a lower pain response after plastic cup than metal cup ventouse delivery (*p <* 0.001), but the pain response was generally lower than expected and they recovered fully within 72 h.

**Conclusions:**

Neonatal pain response is slightly reduced after use of Kiwi OmniCup® versus metal cup ventouse.

**Trial registration:**

Trial was registered under under NCT00488540 on 19th June 2007.

## Background

Childbirth and especially the mode of delivery is a major stressor for the neonate. Approximately 3-20% of all deliveries worldwide are operative vaginal deliveries with a shift from forceps to ventouse deliveries during the last decades [1, 2]. The neonate’s stress and pain response after different types of delivery are difficult to gauge. In a previously published study, we showed that neonates born by assisted vaginal delivery in general had the highest burden of pain compared to those delivered by vaginal birth or caesarean section as reflected by neonatal pain scores and saliva cortisol [3]. Pain response after vacuum extraction was also associated with higher cord blood cortisol levels [4, 5].

The disposable PalmPump® cup, Kiwi OmniCup® (Clinical Innovations Inc., Murray, Utah, USA), introduced in obstetric practice in 1999, seems to be comparable to the conventional metal cup with regard to safety, but has a higher failure rate of 30.1% versus 19.2% [6]. The metal cup resulted in a non-significantly higher rate of substantial head injuries compared to the use of the Kiwi OmniCup® (38 versus 23%) in one study [7] while another study showed no differences in scalp injury [8]. To our knowledge, there is no study examining the pain response after assisted vaginal deliveries using different types of ventouse and/or analysing the relation of the infant’s pain response to the neonate’s head trauma. This study investigates the pain response after metal versus Kiwi OmniCup® ventouse in an observational analysis of a former prospective randomised placebo controlled trial about the analgesic effect of acetaminophen in neonates after operative vaginal delivery.

## Methods

We present a secondary observational data analysis from a former prospective, randomised, double-blind and placebo-controlled, national multicentre study on the effect of paracetamol (acetaminophen) as neonatal pain relief after ventouse deliveries [9]. The study was approved by each local institutional review board in Basel, Bern and Zurich. The trial was registered under www.ClinicalTrials.gov under NCT00488540 on 19th June 2007.

The inclusion criteria were a gestational age of ≥ 35 weeks and a birth weight of at least 2000 g. Exclusion criteria were congenital malformations, administration of opioids to the mother during the last 24 h before delivery, and rotational or failed ventouse delivery. All eligible women were informed about the study during antenatal visits or after entering the delivery ward and were enrolled with a written parental informed consent shortly before or directly after delivery. The obstetric operator, who performed the ventouse delivery, was asked to fill in a questionnaire about the indication and details of the delivery mode, including an account of complications like air suction or “detachment” of the cup from the neonate’s head and the assessment of neonatal head injury directly after each delivery.

Participating neonates were randomised to either treatment with two weight-adapted suppositories of paracetamol (60, 80, or 100 mg per dose) or placebo at 2 and 8 h after delivery, respectively. All neonates, who were transferred directly after delivery to the neonatal unit or left the hospital prior to metabolic testing at day 2 to 3, were considered drop outs. The current observational analysis compared pain response after metal cup versus Kiwi OmniCup® ventouse. Deliveries by forceps or by ventouse using a silicone vacuum device were excluded.

Study outcomes included two validated pain scales, which were assessed at different time points. At first, prolonged pain and discomfort were measured by the EDIN scale (Échelle Douleur Inconfort Nouveau-Né, neonatal pain and discomfort scale) during the first 24 h of life. This pain scale uses five behavioural indicators of prolonged pain: facial activity, body movements, quality of sleep, quality of contact with nurses, and consolability [10]. Each indicator was scored from 0 to 3 by specially trained nurses or midwives at 2, 4, 8, 12, and 24 h. An EDIN score ≥ 5 is considered to indicate significant pain [11]. The primary endpoint was the pain response by EDIN score within 24 h as estimated by the EDIN AUC. Secondly, an acute pain response was then assessed after the standard heel prick for metabolic testing (Guthrie test) at day 2 to 3 of life by 7 behavioural indicator of the Bernese Pain Scale of New-borns (BPSN) with a score of 0 to 3 for each indicator: alertness, duration of crying, time to calm, skin colour, eyebrow bulge with eye squeeze, posture, and breathing pattern [12]. A BPSN of 0–6 represents absence of pain and a score of 9–17 equals a normal response to an acute painful event like capillary blood sampling. BPSN was obtained by nurses directly before and after the heel prick and was also videotaped for scoring by a single reviewer. Correlation (Spearman rank order coefficient) of BPSN scores between nurses and reviewer was 0.798 (*p <* .001) and the rate of agreement (Cohen kappa) to describe a BPSN ≥ 9 was 0.784 (*p <* .001). Additionally to the pain expression scales, salivary cortisol were analysed to measure the biochemical neonatal stress and pain response to acute pain stimulus (heel prick for metabolic testing (Guthrie test). Saliva samples for cortisol levels were collected 5 min before and 30 min after the heel prick by placing a swab under the neonate’s tongue for 2 min and were stored afterwards at −20 °C until analysis at a certified external laboratory for examination by DELFIA® (time-resolved fluoromeric endpoint detection; Wallac, Turku, Finland). Saliva collection was not collected/not available from the centre in Zurich. For the detailed description of the cortisol analysis, see the previously published article by Schuller et al. [3] Additionally, duration of crying was measured after metabolic testing. BPSN, salivary cortisol levels, and scream duration were considered secondary endpoints.

### Statistics

Baseline characteristics of neonates, vacuum variables and outcome/complications are presented stratified for ventouse and suppository content. Continuous variables were tested for differences between the four groups using Wilcoxon signed-rank tests and chi square tests for categorical variables. *P-*values were not adjusted for multiple testing. The primary endpoint was the area under the curve (AUC) for the EDIN score during the first 24 h after birth. EDIN AUC was analysed for an association with ventouse and suppository and their interaction using linear model. Since the original trial was randomised for suppository and not for type of ventouse, the comparison of the latter is of observational character with the risk of confounding. Propensity scores are one option for counteracting such bias. We used boosted logistic regression to correct for imbalances in maternal and birth characteristics and applied to the linear model, using inverse probability weighting (IPW) [13]. The confounding variables—used as predictors for the propensity scores—included centre, analgesia, indication for vaginal operative delivery, operator, gestational age, gender, and duration of first stage of labour. With the exception of centre, covariate balance was significantly improved by propensity score weighting. Therefore, as a sensitivity analysis, centre was included in the propensity score model as was suggested by Heinze & Jüni 2011 [14]. The secondary endpoints, BPSN, saliva cortisol levels, and duration of crying, showed overdispersion and were analysed in a generalised linear model assuming quasi poisson error distribution. All statistical analyses were performed with the statistical software R version 3.1.0 [15]. A level of significance of α = 0.05 was used.

## Results

**Fig. 1:**
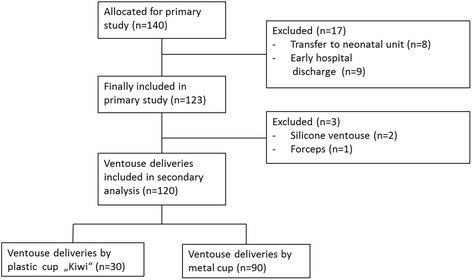
Included and excluded patients of the secondary analysis

A total of 140 neonates were recruited in the primary study. Eight neonates dropped out because of transfer to the neonatal unit and nine neonates left the hospital prior to metabolic testing. Of the remaining 123 infants, we excluded those delivered by a silicone ventouse (*n =* 2) and one delivered by forceps. Finally, 120 neonates were included in the analysis set, 30 of which were delivered by a rigid plastic cup (Kiwi Omnicup®, Clinical Innovations Inc., Murray, Utah, USA) and 90 by a metal cup ventouse (Menox AB, Göteborg, Sweden) (Fig. 1). The baseline characteristics of both groups are shown in Table 1. Infants who were delivered by plastic cup did not differ from infants delivered by metal cup ventouse in respect to gestational age, birth weight, length or head circumference, epidural or spinal analgesia, or arterial umbilical cord pH. Only Apgar scores after 5 min were significantly lower after plastic ventouse. The vacuum variables are expressed in Table 2. Data of moulding, occiput position, or pelvic station (head station relating to the level of maternal ischial spines) were missing. The choice of ventouse strongly differed between the participating centres. Zurich and Basel performed more ventouse deliveries using the metal cup (35/35 and 51/66, respectively). Bern preferred the KiwiOmni® cup (15/19). There were no significant differences in indication for vaginal operative deliveries and traction counts. Air was aspirated in 10% of the metal cup ventouse. The cup detached from the neonates’ heads in two cases using the plastic cup (6.7%) and there was no detachment in the metal cup group. The metal cup provoked more substantial scalp traumas (cephalhaematoma and skin abrasions/lacerations) in 13.2% (12/90) compared to the plastic cup in 6.6% (2/30), but this finding was not significant (*p =* 0.51). There was no case of subgaleal or intracranial haemorrhage and skull fracture.

**Table 1:** Baseline characteristics of neonates

Characteristic	Plastic cup “Kiwi”	Metal cup	p
Gestational age (wk)	40^0/7^ ± 1^1/7^	39^5/7^ ± 1^1/7^	.37
Sex ratio (male:female)	17:13 (56.7:43.3)	50:40 (55.6:44.4)	1.00
Birth weight (g)	3327 ± 440	3432 ± 408	.34
Length (cm)	49.9 ± 2.0	50.4 ± 1.9	.38
Head circumference (cm)	34.9 ± 1.3	35.1 ± 1.5	.55
Arterial cord pH	7.22 ± 0.06	7.25 ± 0.07	.13
Apgar score 1 min	8 (6–9)	8 (8–9)	.17
Apgar score 5 min	9 (8–9)	9 (9–10)	.02
Apgar score 10 min	10 (9–10)	10 (9–10)	.78
Maternal epidural/spinal anaesthesia	74 (83.2)	23 (76.7)	.6

Continuous variables are presented as mean ± standard deviation (SD), except for the Apgar scores, for which median and interquartile range (IQR) are shown. Categorical variables are shown as absolute numbers and percentages

**Table 2:** Ventouse variables and outcome/complications

Variable	Plastic cup “Kiwi”	Metal cup	p
Participating centre			
Bern	15 (50)	4 (4.4)	<.001
Basel	15 (50)	51 (56.7)	
Zurich	0 (0)	35 (38.9)	
Duration of first stage of labour (h)	5.2 (3.7–7.9)	5.1 (3.8–7.5)	.96
Duration of second stage of labour (h)	1.5 (0.9–2.4)	2.1 (1.1–2.8)	.12
Causes for vaginal assisted delivery			
failure to progress in labour	10 (34.5)	43 (47.8)	.33
fetal distress	17 (58.6)	43 (47.8)	
maternal exhaustion	2 (6.9)	2 (2.2)	
others	0 (0)	2 (2.2)	
Number of tractions			
1	5 (16.7)	10 (11.1)	.6
2	12 (40)	50 (55.6)	
3	12 (40)	27 (30)	
4	1 (3.3)	2 (2.2)	
5	0 (0)	1 (1.1)	
Complications			
none	28 (93.3)	77 (85.6)	.01
air aspiration	0 (0)	9 (10)	
cup “detachment”	2 (6.7)	0 (0)	
no information	0 (0)	4 (4.4)	
Severe head injuries			
- skin abrasions/lacerations	1 (3.3)	2 (2.2)	.51
- cephalhaematoma	1 (3.3)	10 (11.1)	
- intracranial haemorrhage	0 (0)	0 (0)	
- skull fracture	0 (0)	0 (0)	
Study treatment			
- paracetamol	18 (60)	43 (47.8)	.34
- placebo	12 (40)	47 (52.2)	

Categorical variables are shown as absolute numbers and percentages. Durations of first and second stage of labour are reported with median and interquartile range (IQR)

**Fig. 2:**
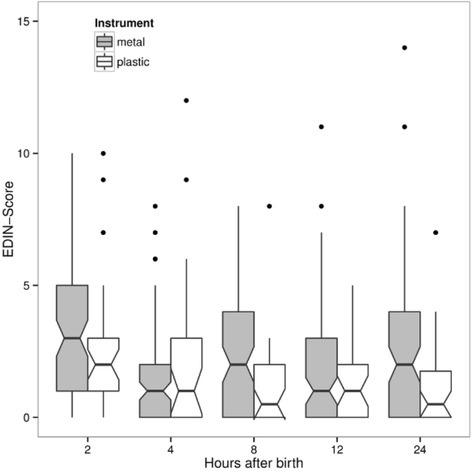
Pain intensity measured by EDIN score at different time points after metal versus plastic ventouse deliveries. The boxes show the interquartile range with the median represented by a horizontal line. Whiskers correspond to 1.5 interquartile ranges

**Fig. 3:**
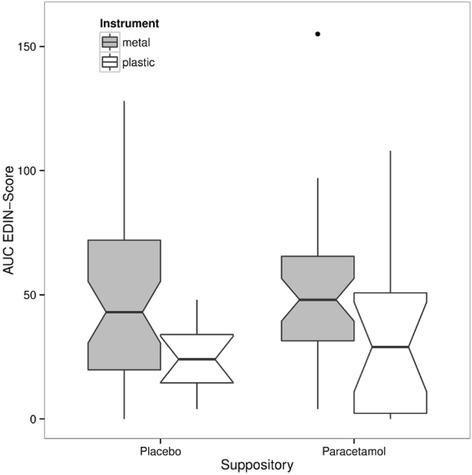
Pain intensity within first 24 h after metal versus plastic ventouse delivery, measured by the area under the curve (AUC) of the EDIN score according to suppository. The boxes show the interquartile range with the median represented by a horizontal line. Whiskers correspond to 1.5 interquartile ranges

The results based on IPW showed a reduction in EDIN AUC by plastic versus metal cup of −23.82 (CI: −36.2 and −11.4, *p <* 0.001; analysis without IPW *p =* 0.02). At each time, there was a tendency of higher EDIN scores after metal cup use with a maximum at two hours after birth (Fig. 2). The EDIN score declined within the next two hours and reached lower values four hours after birth, which was not significantly different in both groups. There was no effect of the suppository on EDIN AUC (*p =* 0.8; analysis without IPW *p =* 0.77) and no interaction of suppository and instrument was found (*p =* 0.63; analysis without IPW *p =* 0.61) (Fig. 3). However, the proportion of neonates with high pain scores (EDIN ≥ 5) was not statistically significantly different at any time (Table 3). The proportion of EDIN ≥ 5 was higher in the metal/placebo versus plastic/placebo group, but the difference was not statistically significant. The effect of maternal analgesia on EDIN score could not be tested because of the high proportion of mothers with epidural/spinal anaesthesia (*n =* 97) versus buscopan only (*n =* 13), other analgesics (*n =* 4), and no analgesia at all (*n =* 4). There was no statistically significant effect of light or severe head trauma on EDIN AUC (*p =* 0.51 and *p =* 0.63, respectively).

**Table 3:** Median and IQR of prolonged pain (EDIN) after delivery, acute pain (BPSN) before/after heel prick at day 2 to 3 of life, and duration of crying after heel prick (*p-*values test difference between all groups)

Variable	Plastic cup “Kiwi”		Metal cup		P
	Placebo	Paracetamol	Placebo	Paracetamol	
EDIN AUC	24 (15–34)	29 (2–50)	43 (20–72)	48 (32–66)	.02
EDIN (median, IQR)					
2 h	2 (1–3)	2 (1–5)	3 (0–5)	3 (2–5)	.34
4 h	1 (0–1)	1 (0–4)	1 (0–2)	1 (0–2)	.55
8 h	1 (0–1)	1 (0–2)	2 (0–4)	2 (1–3)	<.00
12 h	1 (0–2)	1 (0–2)	2 (0–4)	2 (1–4)	.28
24 h	0 (0–1)	1 (0–3)	2 (0–4)	2 (1–4)	.03
EDIN ≥ 5 (%)					
2 h	11.1	34.6	28	28.2	.37
4 h	5.6	15.4	15.9	12.7	.69
8 h	11.1	0	19.5	14.1	.1
12 h	0	7.7	3.7	9.9	.27
24 h	0	7.7	19.5	18.3	.12
BPSN (nurses)					
before heel prick	0 (0–2)	0 (0–1)	0 (0–1)	0 (0–2)	.66
after heel prick	3 (0–7)	5 (4–9)	3 (1–5)	4 (2–7)	.07
BPSN (reviewer)					
before heel prick	0 (0–0)	0 (0–1)	0 (0–1)	0 (0–1)	.7
after heel prick	3 (2–9)	5 (3–7)	3 (2–6)	5 (3–8)	.43
Salivary cortisol levels (nmol/L)					
- before heel prick (*n =* 58)	6.1 (3.7–8.4)	5.1 (4.4–6.8)	3.9 (2.3–7.7)	4.1 (2.5–5.9)	.56
- after heel prick (*n =* 52)	13.2 (8.6–16)	18.9 (4.5–24.2)	8.1 (4.5–25.4)	10.5 (6.6–17.3)	.93
Duration of crying (sec)	3 (0–86)	22 (0–61)	0 (0–23)	16 (0–61)	.27

Salivary cortisol levels could only be measured in 58 out of 85 neonates before and 52 out of 85 neonates after Guthrie testing, respectively, due to insufficient saliva volume for analysis. The evaluation based on IPW showed no significant differences between metal and plastic cup in BPSN before (*p =* 0.86) and after heel prick (*p =* 0.22), salivary cortisol levels before (*p =* 0.56) and after heel prick (*p =* 0.93), or duration of crying after heel prick for metabolic testing at day 2 to 3 (*p =* 0.1), irrespective of the suppository content. The neonates, who received a paracetamol suppository, showed a longer duration of crying, higher cortisol levels, and increased BPSN scores after heel prick in both groups, but these differences were not significant (Table 3).

## Discussion

To our knowledge, this is the first study on neonatal pain response after metal versus Kiwi OmniCup® ventouse deliveries. Neonates delivered by Kiwi OmniCup® ventouse presented a significantly lower pain response in the first 24 h estimated in the EDIN AUC than those delivered by plastic cup ventouse (*p <* 0.001). There were no significantly different EDIN scores ≥ 5, reporting substantial pain at each time point, but the proportion of EDIN ≥ 5 was higher in the metal/placebo versus plastic/placebo group. This observation was not statistically significant, which might be due to lack of power in both placebo groups.

In alignment to the findings by Attilakos et al. [7], we observed a non-significantly higher rate of substantial head injuries after metal versus plastic cup ventouse. Mola et al. [8] found no differences in head trauma between Kiwi OmniCup® and Bird metal cup at all. We found no significant association between substantial or any head injuries and higher EDIN scores. This finding suggests that the reported higher pain response after ventouse delivery might be due to the traction force itself rather than the resulting head trauma. Although an even higher traction level resulting e.g. from head presentation, head station [16] and/or type of device might lead to an increased risk of substantial head injury. In one study by Vacca, the traction force reached up to 11.5 kg (113 *N =* Newton) in 79.8% of successful ventouse deliveries in nulliparous women after Kiwi OmniCup® [17]. In only two cases (1.7%) exceeded the traction force more than 13.5 kg (132 N). In a recently published study [18], the average peak traction force after using the Bird metal cup reached 225 N, which corresponded to a mass equivalent of 23 kg. The traction force after Bird metal cup seems to be substantially higher than those after Kiwi OmniCup® extraction, but a higher proportion of low-cavity and outlet ventouse was included in the study by Vacca [17] compared to the study by Petterson et al. [18]. In a small study from 1979 [19], 216 N (corresponding to a mass of 22 kg) was suggested to be a safe traction force limit for using the 50 mm Malmstrom metal cup. No sensor calculating the traction force was used in our study.

The plastic and the metal cup groups had declining EDIN scores within four hours after delivery reaching steady low scores afterwards. The elevated salivary cortisol levels reported in our previously published study stated that the mode of vaginal operative delivery is the most stressful way of delivery for the infant compared to spontaneous delivery and caesarean section [3]. Although stressful, there might be a regulating analgesic effect by vasopressin during spontaneous and assisted vaginal delivery compared to elective caesarean section [20–24]. Studies comparing the vasopressin levels after vaginal operative delivery and spontaneous delivery are lacking. Interestingly, studies on the stress response (salivary cortisol and duration of crying) after routine vaccination even two months after delivery show an increase in pain response in those infants who had elevated cortisol levels directly after delivery, but the two-month response did not differ by mode of delivery [5]. This suggests that stress at birth could alter the stress regulatory system in the neonate by programming the hypothalamic-pituitary-adrenal axis affecting the pain response at two months of age and might even alter the stress response in later life. This theory however needs to be further elucidated.

There are several limitations to our study. Since the aim of the original trial was not to investigate the effect of different types of ventouse on pain in neonates this comparison is of observational character and groups differ in size. Propensity score methods are currently the best option to reduce potential bias between non-randomised groups, and are superior to “naïve” observational data analysis. However, results have to be interpreted by keeping in mind that potential bias can only be so far reduced as all relevant confounders, i.e. those variables that influence the outcome of interest, are measured. The propensity score weighting was adjusted for the baseline imbalance of the variable centre. Other aspects of practice associated with the preference of the centres for a type of ventouse cup like e.g. experience with the handling of the device could additionally have influenced the results in a not sufficiently acknowledged manner. The strengths of our study are its prospective design, the concomitant use of pain scores and the cortisol measurements. Despite its limitations, our study is the first to investigate the influence of type of ventouse on the neonatal pain response. Our results indicate that neonates delivered by Kiwi OmniCup® ventouse present a slightly reduced pain response within the first 24 h. As this observation might influence further clinical management, it should be interpreted with caution and merits further investigation by a randomised controlled trial.

## Conclusion

In summary, we conclude that infants born by vaginal operative delivery present a slightly reduced pain response after plastic cup versus metal cup ventouse delivery, but the pain response is generally lower than expected and normalises in both ventouse groups within 72 h.

## Abbreviations

AUC: Area under the curve
BPSN: Bernese Pain Scale of New-borns
EDIN scale: Échelle Douleur Inconfort Nouveau-Né, neonatal pain and discomfort scale
IPW: Inverse probability weighting
